# Timing Is Everything. Temporal and Spatial Niche Segregation in *Curculio* spp. (Coleoptera: Curculionidae) Associated with Oak Trees

**DOI:** 10.3390/insects12080687

**Published:** 2021-07-31

**Authors:** Michał Reut, Mariusz Chrabąszcz, Hanna Moniuszko

**Affiliations:** 1 Section of Applied Entomology, Department of Plant Protection, Institute of Horticultural Sciences, Warsaw University of Life Sciences—SGGW, Nowoursynowska 159, 02-776 Warsaw, Poland; hanna_moniuszko@sggw.edu.pl; 2 Department of Ecology, Biogeochemistry and Environmental Protection, Faculty of Biological Sciences, University of Wrocław, Kanonia 6-8, 50-328 Wrocław, Poland; mariusz.chrabaszcz@uwr.edu.pl

**Keywords:** competition, weevils, ecology, phenology, *Quercus*, reproduction

## Abstract

**Simple Summary:**

Acorn weevils from the genus *Curculio* (Curculionini) represent a group of species developing in fructifications of plants. In Poland, three different species of those beetles develop on the same host plant and use the same resources, which can lead to increased competition. We tested preferences of weevil females towards acorn size chosen for oviposition as well as reproduction time and other characteristics of their breeding behavior. The results clearly indicate that particular species prefer acorns of different masses and breeds in different periods corresponding to different stages of fruit growth. In other words, this enables weevils to pass each other during reproduction. Furthermore, one of the studied species varies distinctly also in terms of the number of laid eggs and the manner of locating them within the acorn tissues. Our observations support the hypothesis that niche partitioning among the species appears to be occurring when the resources—acorns, are limited. The work helps to understand how multi-species communities of insects cope in order to survive in a shared habitat.

**Abstract:**

Oak seed predatory weevils occurring in Poland are prone to increased interspecific competition due to the limited number of *Quercus* species, compared to southern Europe, in which they can develop. Therefore, analyses on the preferences of three weevil species for acorn sizes chosen for reproduction, as well as on reproductive period duration, were performed. Cafeteria-type experiments were set for females of three species associated with one oak species. Females were allowed to choose and oviposit in acorns of different sizes and growth stages. Research revealed statistically significant differences between the masses of acorns chosen for oviposition by females of *Curculio glandium* (the biggest), *C. pellitus* (medium), and *C. venosus* (the smallest). Studied weevils also differed in terms of the beginning of the reproductive period, which corresponded with the increasing mass of growing acorns. Moreover, *C. glandium* was observed to be the only species to perform radial egg galleries and lay a considerably higher and varied number of eggs. The results support the hypothesis of a strategy aimed at reducing interspecific competition between *Curculio* spp. in terms of limited host plant species number.

## 1. Introduction

Similar food and microhabitat requirements typically lead to the overlapping of niches. This was thoroughly described in insects representing seed predators [1,2]. In many cases, overlapped niches combined with limited resources trigger strong competition between species, as is the case in weevils [3].

European, acorn-associated representatives of the *Curculio* L. genus (Coleoptera: Curculionidae: Curculionini) inhabit several oak species in the southern part of the continent [4] whereas in central Europe (including Poland), these insects live only on two *Quercus* hosts—commonly occurring pedunculate oak *Quercus robur* L. and rare sessile oak *Q. petraea* (Mattuschka) Liebl. In conditions of eastern Europe, the occurrence of *Q. petraea* is much more rare, which means that *Q. robur* is the main host plant for at least three *Curculio* species, *C. glandium*, *C. pellitus*, and *C. venosus*. In the site under consideration (park of the Warsaw University of Life Sciences), only one species (*Q. robur*) is planted. Other European curculionids are associated with generative organs of birches, hazels, and willows, as well as to a variety of herbaceous and cultivated plants.

Acorns, being food for larval instars, are usually a limited resource due to various factors, with the first one being a masting. It is a regularly recurring phenological phenomenon observed in many woody species of plants, i.e., trees and shrubs [5]. In the case of oaks, it is clearly visible in an acorn production that is highly variable—in mast years, fruits are present in high numbers, densely covering the grounds, while in non–mast years, there are almost no acorns available for animals [6]. Masting is potentially characterized by the highest impact on seed predators, which include acorn weevils [7,8]. Climatic factors, like a drought, can influence acorn production as well and thus affect weevils [9]. Vertebrate feeding on acorns is another factor limiting the accessibility of this resource for insects [10]. Moreover, commonly occurring and numerous large and small vertebrates (e.g., wild boars, roe deer, deer, and rodents) can directly limit weevil population by consuming acorns infested by developing larvae [11,12].

All above-mentioned factors lead to a supposition that a strong competition within *Curculio* species inhabiting oaks and acorns, especially in non-mast years, can be expected. The latter was investigated during the research by Bonal et al. [13] in which co-occurring *Curculio elephas* (Gyllenhal) and *C. glandium* Marsham associated with *Q. ilex* L. and *Q. humilis* Lamarck in Spain were tested for the potential morphological adaptations, associated with a rostrum length, to acorns of a different size.

As previously stated, in Poland and Baltic countries, three *Curculio* species feeding on oaks were observed—*C. glandium*, *C. venosus* (Gravenhorst), and *C. pellitus* (Boheman) [14]. The fourth species, *C. elephas*, can be rarely found, but it seems to be more associated with *Castnea sativa* trees [15]. Among listed weevils (*glandium, pellitus*, and *venosus*), *C. glandium* is characterized by the longest snout, i.e., rostrum; it is also not closely related to the other acorn weevils but rather to a hazelnut weevil, *C. nucum* L. [16]. Nonetheless, these species are known to coexist in the same environment [17], but the amount of data on their biology is scarce. Recent studies regarding the representatives of the Curculionini tribe inhabiting the natural environment are aimed rather at taxonomical and phylogenetic issues, e.g., [18,19,20].

In this research, we aimed to better understand ecological differences related to reproduction between *C. glandium*, *C. pellitus*, and *C. venosus* associated with the pedunculate oak, *Q. robur*, in the Municipal Park in Warsaw (Poland), based on laboratory experiments combined with field observations. We hypothesize that due to the use of the same resources, (1) females of different species show preferences towards acorns of a certain mass and size, which is related to starting their reproductive period at different times; (2) species differ in terms of laid eggs number; (3) there is a pattern of an even egg distribution within galleries drilled by *C. glandium* females.

## 2. Materials and Methods

### 2.1. Experiment 1—Ovipositional Strategies among Curculio Species

During the years of 2013 and 2014, we searched for representatives of various *Curculio* species inhabiting *Q. robur* trees in an old park at University Campus in Warsaw, Poland (52°12′ N 21°00′ E). The study site covers a park located on the northern side of the Nature Reserve ‘Skarpa Ursynowska’ with the native and non-native mostly deciduous tree species, including more than 50 oaks, at ages varying from 30 to over 100 years old. Most of the park has a lot of open space and tree branches grow low, which makes easy access to the acorns. During the field investigation, we observed initiation of a breeding season of three different *Curculio* species. Observations were carried daily (except for rainy and very windy days) from the end of May until the end of August. Whenever we found any acorns with weevils in copula for the first time, we noted the species, time and weather conditions. Until the end of June, 34 females of *C. venosus* and 19 of *C. pellitus* were caught and taken for a laboratory survey. Fifty *C. glandium* females were collected in June, however, this species is possible to be caught in hundreds. The ratio of captured males (needed only for fertilization of females) was similar. Beetle species were determined with the use of the key by Smreczyński [21].

Later on, we kept individuals of three species in laboratory conditions until they started mating and laying eggs. Weevils were cultured in groups consisting of both sexes belonging to the same species. Initially, insects were kept only with a piece of an apple as a source of food and water, without acorns as ovipositional sites. During the experiment a single female of each *Curculio* species was placed in a plastic container (13 cm × 9 cm × 3.5 cm) with access to acorns of different sizes (placed 6 cm apart from each other). We set the following fruit size-mass categories offered to each female, defined as: I. small acorn—weight under 1 g; II. medium—weight between 1 and 2 g; III. big acorn—weight above 2 g. Each time acorns were harvested earlier in the morning, on the same day as the experiment was carried out. They were weighted just before the experiment. In addition, a piece of an apple was provided. The lighting source was an LED lamp with additional red light for nighttime. Photoperiod was set each week to resemble the natural day and night cycle occurring in July and August (when *Curculio* species lay eggs). Females were let to oviposit for a period of 48 h.

Insects were recorded using an Internet Protocol surveillance camera (Day/Night megapixel IP camera, Novus, Poland), so we knew exactly which acorn was chosen for drilling and oviposition. After 48 h, acorns were carefully dissected and checked for the presence of eggs. The number and localization of eggs were noted.

The normality of the data distribution was checked using Shapiro–Wilk’s test, which proved the non-normality of the distribution. In consequence, Kruskal–Wallis’ non-parametric ANOVA and a post-hoc Dunn’s test were performed to assay:

- differences between masses of acorns chosen by particular *Curculio* spp. females;
- differences in the number of eggs laid by *Curculio* spp. females.

### 2.2. Experiment 2—Distribution of Eggs and Egg Galleries in Curculio Glandium

To unveil how *C. glandium* females distribute eggs within the egg channels, and also to see the structure of such galleries, in August 2013, a total of 120 fallen, fresh acorns from the field site (University Campus, Warsaw, Poland) were collected and checked for the markings (tiny holes and discoloration of the outer skin) indicating the presence of egg galleries. Surfaces (pericarps) of these acorns were carefully removed with a surgeon blade so as not to damage the laid eggs. Because in the case of acorns from the field, we were not able to verify how many different females laid eggs to a specific fruit, another experiment was carried out in a laboratory.

In August 2013, we let 50 field-collected females of *C. glandium* (also used in Experiment 1 during the end of July) drill in young, uninfested acorns, i.e., selected ones, devoid of above-mentioned beetle activity traces. Every female was kept separately for 24 h with access to an acorn to make sure that all eggs were laid by only one female. A piece of apple was provided as a food and water source. Then all acorns were cut open, and the number of eggs and channels (empty or with an egg at the end) were counted. Photo documentation was carried out using a digital camera (Canon 50D with 100 mm macro lens).

**Figure 1:**
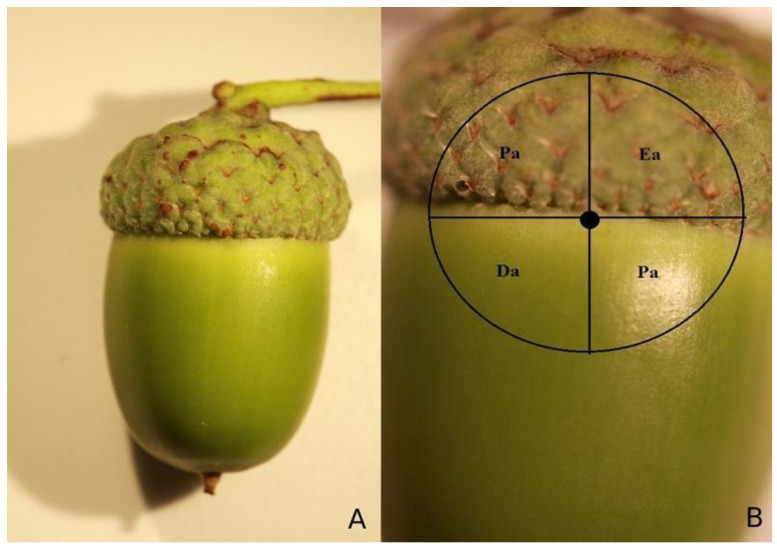
Scheme of egg distribution in an acorn: (**A**) general view of a fruit, (**B**) close up with marked egg areas: *Ea*—egg area, where the first egg was laid; *Pa*—proximate area, i.e., locations close to laid egg; *Da*—distant area, which was more than 45° from laid egg.

To check the possibility of females ovipositing in a specific manner (e.g., locating the next egg far from the previous one), we continued observations on their behavior and took notes, where the second eggs were laid by the same female (*n* = 20). In this case, an IP camera was used to record how the ova were distributed by females. The recordings took 24 h per female, then were analyzed, so the locations of each egg were possible to find. Afterwards, surfaces of all acorns were carefully removed with a surgical blade. We checked if the positions of found eggs were the same as those on recordings. These locations were noted down following the scheme shown in Figure 1. The hypothesis, whether there is a pattern of egg distribution, was tested with *χ*^2^ test—we have compared the position of the second egg with positions that would be theoretically random (equal egg distribution).

The results of both experiments were analyzed in Statistica software (v. 13.0, StatSoft Inc., Tulsa, OK, USA).

## 3. Results

### 3.1. Ovipositional Strategies among Three Curculio Species

**Figure 2:**
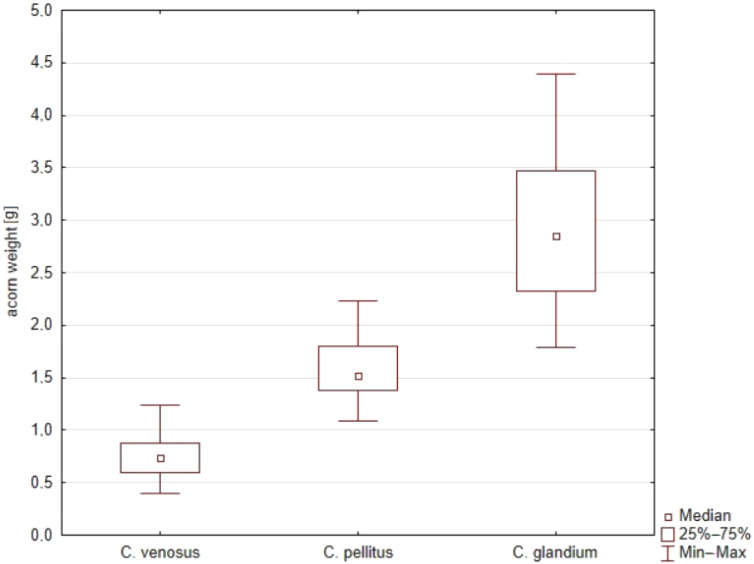
Significant differences between masses of acorns chosen for oviposition by females representing three *Curculio* species (Kruskal–Wallis’ test, *p* < 0.05).

The general characteristics of acorn selection by *Curculio* spp. females are shown in Table 1. Generally, females of *C. venosus* were rarely interested in drilling into the heaviest acorns. Their eggs were found in the smallest fruits, with weight <1 g (median 0.741 g). Females of *C. pellitus* preferred bigger acorns, usually with weight around 1.5 g (median 1.519 g), sometimes choosing the biggest ones. *Curculio glandium* females, in turn, drilled in all kinds of acorns but laid eggs only into the biggest ones, with a mass over 2.5 g (median 2.852 g). Kruskal–Wallis’ test revealed a significant (post-hoc, *p* < 0.05) difference in the selection of acorns in terms of weight by females of the three species. Dunn’s post-hoc test showed that the lightest acorns were selected significantly more often by female *C. venosus* (post-hoc, *p* < 0.05) than by females of the other species. *Curculio glandium* females selected the acorns of the significantly largest masses (post-hoc, *p* < 0.05). The above differences are illustrated in Figure 2.

General characteristics of the number of eggs laid by *Curculio* females are shown in Table 2. In the laboratory trials, most of the investigated females of *C. venosus* laid single eggs (*n* = 25) into acorn cupule. Rarely it was two (*n* = 8) or three (*n* = 1) eggs. *Curculio pellitus* females also laid only one egg (*n* = 5), but usually, it was more—two (*n* = 9) or three (*n* = 5).

**Figure 3:**
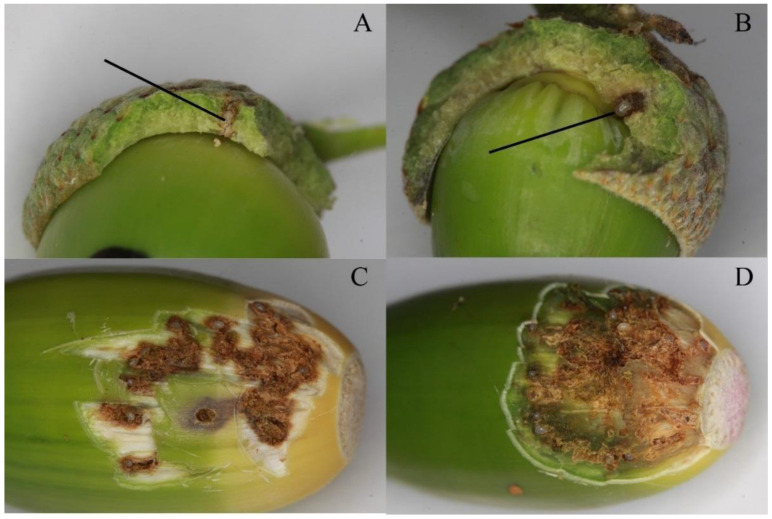
Typical view on oviposition sites in acorns infested during the first experiment: (**A**,**B**) *C. venosus*; (**C**,**D**) *C. glandium*. *Curculio venosus* usually lays one egg per acorn (marked with an arrow), always into cupula. Contrary, *C. glandium* can oviposit few (even 10 or more) eggs into one acorn, just below the outer skin, and not into the cupula.

Both species never drilled egg galleries as ones observed in acorns infested by *C. glandium* (Figure 3C,D). The latter species behaved differently in many aspects. Females of *C. glandium* laid varied numbers of eggs, i.e., one (*n* = 3), two (*n* = 8), three (*n* = 7), four (*n* = 18), five (*n* = 7), six (*n* = 4), seven (*n* = 1), up to eight (*n* = 2); numbers of laid eggs seemed to increase as acorns matured and less were available for weevils (M.R. personal observation). Kruskal–Wallis’ test showed a significant (*p* < 0.05) difference in the numbers of eggs laid by females of the three *Curculio* species, with *C. glandium* laying significantly more eggs than other two species (post-hoc, *p* < 0.05). No significant difference (post-hoc, *p* > 0.05) was observed between the number of eggs laid by the females of *C. venosus* and *C. pellitus*.

**Figure 4:**
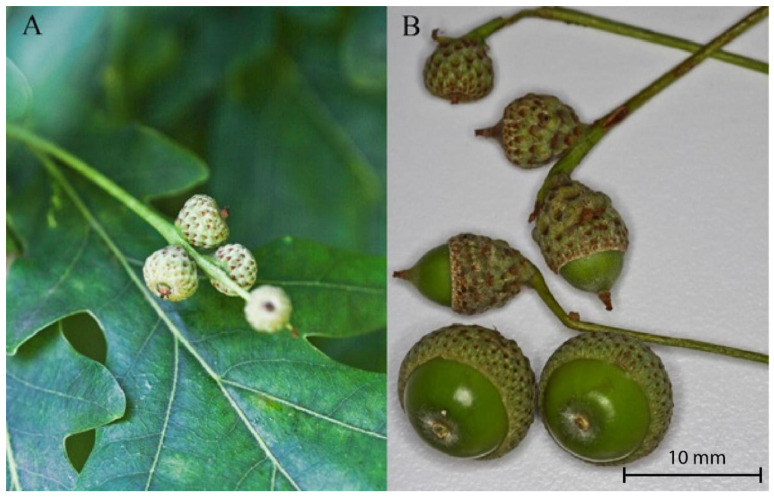
Acorns in the early stage of growth: (**A**) fruits in June are usually very small—taken on 16 June 2014, (**B**) acorns during growth vary in size and shape between trees—photo taken on 8 July 2014.

The first copulating pair of *C. venosus* was observed in the laboratory at the beginning of July (3 July 2014); at this time, acorns were still small (Figure 4). This corresponded with insects’ preferences (see part above). The second week of July was a time when we noted *C. pellitus* starting to breed—first in the laboratory (11 July 2014) and later in the field (14 July 2014). The last species to breed was *C. glandium*, mating couples of which were found only since the end of July (22 July 2014).

### 3.2. Distribution of Eggs and Egg Galleries in Curculio Glandium

**Figure 5:**
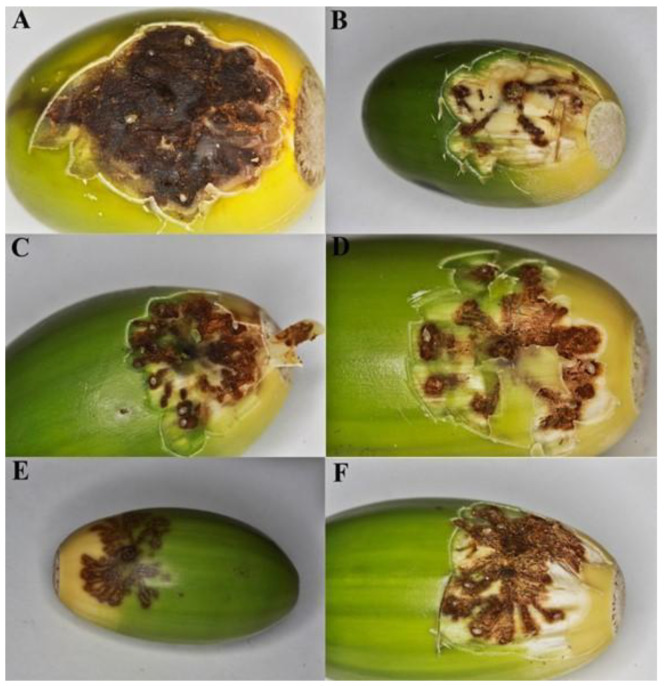
Egg galleries created by *C. glandium* females: (**A**–**D**) eggs placed at the end of the channels; all channels start from the same point, resembling a star in shape; many empty channels can be seen, (**E**,**F**) the same acorn before and after surface removal.

Out of 120 field-collected acorns, 89 contained more than four *C. glandium* eggs in galleries. The egg galleries were excavated in a radial pattern—multiple egg channels drilled from the single point (Figure 5).

All of the 50 new acorns prepared for the 50 females (also used in Experiment 1) to oviposit in, were infested during the second experiment. We did not find an acorn with less than four egg channels. Usually, one egg was found in every five channels (min. *n* = 4, max. *n* = 8, med. 5.66). No egg gallery was found not containing a single egg. All the galleries were located just below the surface (pericarp) of acorns. It took many hours for a female to perform such a gallery, and so females started moving around one point intensively.

Based on observation of 20 *C. glandium* females, we found that the period between the laying of the first and the second eggs was on average 1.02 h. (max. = 2.47 h., min. = 0.21 h., s.d. = 1.18). Females usually spent 1.08 h. on drilling since laying the second egg until the third appeared (max. = 2.25, min. = 0.28, s.d. = 1.12). The time between the third and the fourth laid egg was on average 2.12 h. (max. = 3.25, min. = 0.29, s.d. = 1.05).

Females placed the second eggs in a random manner, not only in a proximate area (*p* > 0.05, *χ*^2^ = 0.313). The third (*p* > 0.05, *χ*^2^ = 0.055) and the fourth (*p* > 0.05, *χ*^2^ = 1.73) eggs were also placed in various locations, thus proving the third hypothesis wrong.

## 4. Discussion

We investigated temporal and size-related niche segregation in three-species *Curculio* community infesting *Quercus robur* acorns in an old municipal park in Warsaw, Poland. Based on observations made, both in the field and laboratory conditions, we noticed ecological and behavioral differences between the studied species. All of them are known to develop from pupae into adult form in early spring and are quite commonly found on various trees in May and June. The three were also reported to breed in the summer; some authors state that this occurs during the same period of time [17]. According to our observations they do not, having different temporal and resource niches.

Females of *C. venosus* were observed as the first of all three species to copulate and oviposit into the youngest acorns. They started in the first week of July, with the first observation noted on 1 July 2013. Contrary to observations by Pellison et al. [17], none of the species was ovipositing in June as the acorns were then too small to enable larval development (Figure 4). We noticed that another weevil species, *C. pellitus*, began reproduction around two weeks later—the first copulating pairs were observed in the middle of July. Finally, at the end of July, the last species started breeding season—mating pairs of *C. glandium* were found at this time for over three years of observations (2012–2014), i.e., including a season related to another project. Existing data on *Curculio* species living in different regions showed that tropical weevil species tended to be host specialists more than generalists, like weevils from temperate areas. For example, *Curculio* beetles from Nicaragua are characterized by higher alpha, beta and gamma diversity than species studied in California, despite similar host richness in both locations [22]. Also, recent data from China give an insight into a host specialization of *Curculio* weevils inhabiting subtropical evergreen broad-leaved forests [23].

During the study, we also found that the three weevils significantly differed in terms of choice of utilized fruit mass. Acorns differed in mass, size, and shape during growing (Figure 4). We conducted behavioral cafeteria-type experiments wherein females of studied species were offered three mass and size categories of acorns to choose from (see Methods: Experiment 1) to oviposit. *Curculio* females’ mass preferences seemed to be related to their temporal niche. Females of *C. venosus*, which bred first, preferred the smallest and the lightest acorns—with a median weight of 0.741 g. Conversely, females of *C. glandium*, breeding mainly in August and September, showed a tendency to lay eggs into the heaviest acorns—median weight of which was 2.852 g. Finally, *C. pellitus*, with the median mass of selected fruits being 1.519 g, was located in between. Having the same host requires an optimal timing for weevils to reproduce.

We also revealed that *C. glandium* females laid significantly more eggs than the other two weevils, which can be considered accordant to the already described preferences of this species for the most grown acorns, providing enough space for an increased number of eggs. Such behavior is with the highest probability also related to the food resource available for larval instars. According to studies on *C. elephas* [24], acorn size affects the growth and development of larvae. If females *C. venosus* laid more eggs into small acorns, as they breed early in the season, it would lead to starvation of some larvae.

According to our observations based on video recordings, none of the females of *Curculio* spp. deposited marking pheromone after the oviposition. Marking pheromones seems to be rather common in Curculionidae and were especially well studied in pests. For example, a pepper weevil, *Anthonomus eugenii*, is known for host marking behavior, and the composition of its pheromone has been recently examined [25]. Releasing the substance right after the oviposition lets females avoid overcrowding in ovipositional sites caused by adding the eggs of the female and/or other females’ ova to a given site [26].

Another interesting difference in behavior was a phenomenon observed only in *C. glandium* and was related to reproduction as well. Based on our experiment in the laboratory, we found that a single female created more egg tunnels than needed for oviposition. Usually, a female makes a few attempts to lay eggs until finally leaving the acorn (M.R. personal observation). After dissecting various acorns, we discovered multiple tunnels created by a single female, but only a few eggs.

To our knowledge, excavating such galleries is a very unique behavior in *Curculio* females as none of the closely related *Curculio* species was reported to lay eggs in such a manner. For comparison, observations on acorns infested by *C. venosus* and *C. pellitus*, indicated that these species had not created egg galleries; for example, *C. nucum*, developing on a hazel (*Corylus avellana*), does not create egg galleries (M.R. personal observation). Only in one well-studied related *Curculio* species, *C. camelliae*, authors documented the ovipositional site as a simple hole in the fruit [27]. In the whole Curculionidae family, only wood borers of Scolytinae are known to make galleries [28], although in Scolytinae the gallery is not made by a female but rather by foraging larvae. As the tunnels are at least partly visible from the outside, excavating many tunnels in a gallery might work as camouflage against parasites, for example, the ichneumon wasp, *Scambus colobatus* [29]. Random distribution of eggs in galleries would also support this possibility.

The above-described differences in breeding characteristics are potentially reflected also in phylogenetic relations between the three species. These insects belong to the same tribe of Curculionini, but *C. glandium* belongs to a different group than *C. venosus* and *C. pellitus*, which are closely related and represent the same group as *C. elephas* [30,31,32]. In fact, *C. nucum* developing on hazel, *Coryllus avellana*, is the closest relative of *C. glandium*.

Since the rostrum length co-evolved with the size of host-crops in *Curculio* genus [16,31], it is worthwhile to highlight that *C. glandium*, which has one of the longest rostrum among closely related species feeding on acorns [16], excavates the egg galleries not very deep inside the fruit. In fact, the thickening of some parts of acorns is known as a counter adaptation against acorn weevil infestation [33,34]. It should be unveiled in further studies why females of the mentioned species possess such a long rostrum, despite excavating only acorn surfaces.

## 5. Conclusions

In conclusion, the results we obtained support the hypothesis on different temporal and spatial niches being selected by particular *Curculio* species. The first one to reproduce, *C. venosus*, prefers the smallest acorns; *C. pellitus* chooses medium-sized fruits, and the last one to start breeding, *C. glandium*, drills into the most grown acorns. The strategy allows insects to pass each other while using the same resource. *Curculio glandium* is also the only species to perform radial egg galleries under acorn pericarp and lays a considerably higher number of eggs than two other species. The latter differences may be reflected in phylogenetic relations between these representatives of Curculionini tribe, with *C. venosus* and *C. pellitus* being more related with each other than with *C. glandium*.

## Figures and Tables

**Table 1:** Sample sizes of *Curculio* spp. females, minimal, maximal and median values, and quartile range of acorn masses (in grams) chosen by the particular species. Significant differences are marked with different letters. Bold fonts means the main variable upon which statistical evaluation is based.

Species	N	Min	Max	Median	Quartile Range
*C. venosus*	34	0.394	1.241	**0.741 ^a^**	0.278
*C. pellitus*	19	1.083	2.231	**1.519 ^b^**	0.425
*C. glandium*	50	1.782	4.388	**2.852 ^c^**	1.137

**Table 2:** Sample sizes, minimal, maximal and median values, as well as the total number of eggs laid into acorns by particular species. Significant differences are marked with different letters. Bold fonts means the main variable upon which statistical evaluation is based.

Scheme	N	Min	Max	Median	Sum
*C. venosus*	34	1	3	**1 ^a^**	44
*C. pellitus*	19	1	3	**2 ^a^**	38
*C. glandium*	50	1	8	**4 ^b^**	194

## Data Availability

All data is available at: https://doi.org/10.6084/m9.figshare.15052476; (accessed on 26 July 2021).
